# Hearing Preservation Rates After Cochlear Implantation Using Soft Surgery Techniques

**DOI:** 10.1002/lary.70664

**Published:** 2026-06-11

**Authors:** Tina C. Huang, Scott Lunos

**Affiliations:** ^1^ University of Minnesota Minneapolis Minnesota USA

**Keywords:** cochlear implantation, hearing preservation, soft surgery

## Abstract

**Objectives:**

To determine if hearing preservation rates after cochlear implantation varied over time with soft surgery techniques.

**Methods:**

The hearing preservation rate over time in adult cochlear implant patients over 10 years was analyzed. The association between hearing preservation rate and sex, age, perioperative steroid dose, and type of electrode was analyzed. Low frequency hearing preservation was analyzed separately.

**Results:**

There was no statistical difference with sex, age, or type of electrode. There was a statistically significant improvement in hearing preservation with a higher dose of perioperative steroid on univariate analysis (36.4% vs. 15%). There was a small but significant correlation (*r* = 0.22) between improved rate and later year of implantation. However, no statistically significant correlation was identified for any factor on multivariate analysis. Date of surgery and steroid dose were correlated and, when the correlation was modeled, there was still no correlation with hearing preservation. For low frequency hearing preservation, a higher dose of perioperative steroids and time were also found to be significant on univariate analysis (33% vs. 14.8%). Multivariate analysis did not show significance for any factor, but the date of surgery and steroid dose were noted to be correlated. Model based on the correlation showed that date of surgery and steroid dose were significantly correlated with hearing preservation (*r* = 0.21).

**Conclusions:**

There was a small but significant correlation between the hearing preservation rate over time with the use of soft surgery techniques including a significant difference with the use of a higher steroid dose.

**Level of Evidence:**

3.

## 1 Introduction

Cochlear implant technology has advanced from single channel electrodes to multi‐channel electrodes. The average speech understanding score for an implanted patient is now in the 70% range with many recipients, particularly young children, scoring in the high 90s. This improvement in performance has led to a widening of the criteria for those qualifying for a cochlear implant.

Initially, only patients with profound bilateral hearing loss underwent cochlear implantation due to expected complete loss of residual hearing. However, Hodges et al. reported that 21 out of 40 patients who had undergone cochlear implantation had recordable hearing in one frequency between 500 and 2000 Hz [1]. Given the tonotopic orientation of the cochlea, the possibility of preserving lower frequency hearing was theorized. This led to Gantz and Turner testing a hybrid electrode that was extremely thin and shorter than standard electrodes with the goal of preserving low frequency hearing [2]. All six subjects had preservation of low frequency hearing and improvement in speech understanding when wearing both the cochlear implant and hearing aid plus a hearing aid in the non‐implanted ear. They showed that use of both a hearing aid and a cochlear implant, termed electro‐acoustic stimulation (EAS), in the same ear was feasible. Once it became apparent that hearing preservation (HP) was possible, research accelerated in this field.

There are multiple ways of reporting HP. In 2013, a large multi‐national group, the HEARRING group, proposed a cochlear implant HP classification model which takes into account the preoperative hearing levels as many people receiving a cochlear implant have no testable hearing prior to implantation [3]. This alleviates the result of a patient appearing to have excellent HP because the postoperative and preoperative hearing thresholds were unchanged due to a floor effect.

This study aims to evaluate the HP rates at an academic center over a 10‐year period to assess if a trend towards better rates occurred over time with the advent of soft surgery technique using the HEARRING model. The influence of other factors such as steroid administration and electrode type was also examined.

## 2 Materials and Methods

This study was approved by our institutional review board. A retrospective chart review was performed of patients older than 18 who underwent cochlear implantation between January 1, 2010 and December 31, 2020 using CPT code 69930. Patients with audiometric data from outside institutions were included. Exclusion criteria included revision surgery, lack of preoperative audiometric data and lack of postoperative audiometric data.

### 2.1 Audiometric Data

Audiometric threshold testing of either unmasked or masked air conduction was used for the preoperative and postoperative threshold evaluation. Thresholds at 250, 500, 750, 1000, 1500, 2000, 3000, 4000, 6000, and 8000 Herz (Hz) were recorded. Pure tone average (PTA) was calculated using the same frequencies for both the preoperative (PTApre) and postoperative (PTApost) audiograms. Low frequency HP was calculated using 250 and 500 Hz.

### 2.2 Surgical Technique

Implantation was performed by three surgeons, with one retiring mid‐2019. All implants were placed through a posterior tympanotomy approach. Cochlear entry was via a cochleostomy, with phasing in of round window insertion between 2012 through 2013, with almost all approaches through the round window by 2014 unless there was difficulty accessing the round window. The speed of insertion, though not specifically measured, increased from less than 30 s to more than 1 min from 2017 through 2020. Administration of a preoperative dose of dexamethasone began in 2013 with no postoperative steroids. The cochlear opening was packed with fascia, with no tissue glue or bone cement.

### 2.3 Implants

Cochlear and Advanced Bionics devices were used throughout the study period with a small number of MED‐EL devices. The electrode types were classified as either straight/lateral wall arrays or perimodiolar/mid‐scala arrays. Manufacturer choice was patient driven. The surgeons did not influence the choice of manufacturer unless there were anatomic considerations. Electrode type was chosen by the surgeon.

### 2.4 Statistical Methods

Statistical analyses were performed using SAS V9.4 (SAS Institute Inc., Cary, NC).

The Wilcoxon rank sum test was used to compare HP for sex, electrode type, and steroid dose. Spearman's rank order correlation was used for comparison of HP over time and age. *p*‐value < 0.05 was considered to be statistically significant. Analyses were performed using all thresholds as well as thresholds less than 100 dB to account for patients with thresholds at the maximum output of the audiometer or untestable. Additional analyses were performed for low frequency thresholds only (250 and 500 Hz).

## 3 Results

### 3.1 Demographics

A query based on CPT code 69930 returned 562 procedures from 477 patients with 85 patients receiving bilateral, either simultaneous or concurrent, implants. Of the 477 patients, 264 were identified as meeting criteria. Of the 213 excluded patients, all were due to lack of audiometric data. The 264 patients had a total of 316 implanted ears. The group consisted of 124 males (47%) with a mean age at the time of the first implant of 57.7. The mean time between surgery and the first postoperative audiogram was 27 days. The results are summarized in Table 1.

**TABLE 1:** Demographic data.

Number of patients		264
Number of implanted ears		316
Sex (%)	Male	124 (47)
	Female	140 (53)
Age (mean, range)		57.7 (18–92)
Days to postoperative audiogram (median, interquartile range)		27 (22–195)
Electrode (*n* 312) (%)	Straight	234 (75)
	PM	78 (25)
Dexamethasone (*n* 304) (%)	< 8 mg	109 (35.9)
	8–10 mg	195 (64.1)

### 3.2 Total Group HP

HP score was calculated using the HEARRING group formula and classified according to the group's classification system. The formula aims to eliminate the effect of preoperative hearing on preservation results, taking into account maximum detectable thresholds to give a value that is the relative change of percentage of hearing loss. Table 2 gives the maximum PTA for each frequency.

$$S=\left[\;1–{\left(\left({\mathrm{PTA}}_{\text{post}}-{\mathrm{PTA}}_{\mathrm{pre}}\right)/\left({\mathrm{PTA}}_{\max}-{\mathrm{PTA}}_{\mathrm{pre}}\right)\right)}^{*}\;100\right]\;\left[100\%\right].$$

**TABLE 2:** Maximum detectable hearing (mdh) measurable for each frequency.

Hz	125	250	500	750	1000	1500	2000	3000	4000	6000	8000
mdh	90	105	110	120	120	120	120	120	115	100	95

The HP values are also classified into complete HP, partial HP, minimal HP and loss of hearing/no hearing (Table 3). Higher values indicate better HP. The HP for all ears was 28.3 with a 95% confidence interval (CI) (19.4, 37.3) and standard deviation (SD) 80.9.

**TABLE 3:** Scale for HP classification.

Percent of residual hearing	Classification
> 75%	Complete HP
> 25%–75%	Partial HP
0%–25%	Minimal HP
No measurable hearing	Loss of hearing/no hearing

When this was broken down into categories, 20 (6.33%) had complete HP, 101 (31.96%) had partial HP and 195 (61.7%) had minimal HP (Table 4). Of the minimal HP group, 110 (56.41%) had no measurable hearing which was 34.81% of the total ears. When looking at PTApre, 12 (3.8%) ears had no measurable hearing. Results were calculated for PTApre < 100 (*n* = 226). The mean HP in this group was 25.6 with 95% CI (22.0, 29.1) and SD 27. This was not significantly different. HP for low frequency hearing showed the same mean HP and was also not significantly different.

**TABLE 4:** Hearing preservation classification for all ears.

Minimal (%)	195 (61.7)
Partial (%)	101 (32)
Complete (%)	20 (6.3)

### 3.3 Demographic

There was no significant difference in HP between sexes in either the entire data set, PTApre < 100 or low frequencies. Female mean HP was 24.1 versus 33.9 with a *p*‐value 0.4485 and in the PTApre < 100, female mean HP was 24.5 versus 27.2 with a *p*‐value 0.5930 (Table 5). There was no correlation with increasing age with Spearman correlation coefficient *r* = −0.08 in the total group and *r* = 0.05 in the PTApre < 100 group. These *r* values are not statistically different than 0.

**TABLE 5:** Mean hearing preservation for sex, electrode type and dexamethasone dose.

		All ears	PTApre < 100 dB	Low frequency
Sex (SD)	Male	33.9 (113.10)	27.2 (29)	31.6 (108.8)
	Female	24.1 (33.5)	24.5 (25.3)	21.8 (30.9)
Electrode	Straight	32.8 (92.6)	26.5 (27.2)	29.3 (88.5)
	PM	19.4 (25.6)	22.5 (25)	17.8 (25.7)
Dexamethasone dose^a^	< 8 mg	15.0 (21.8)	18.1 (23.3)	14.8 (22.9)
	8–10 mg	36.4 (100.7)	29.6 (27.9)	33.0 (96.3)

^a^ Wilcoxon rank sum test *p* = 0.0001 (Statistically significant at 0.05 level).

### 3.4 Electrode Type

Comparison between straight and PM electrodes also did not produce a significant difference in either group. The mean perimodiolar HP in the total group was 19.4 versus 32.8 with a *p*‐value of 0.16 and in the PTApre < 100 group, the mean perimodiolar HP was 22.5 versus 26.5 with a *p*‐value of 0.52 (Table 5).

### 3.5 Dexamethasone Administration

In 2013, a single dose of preoperative dexamethasone began to be used. The dose was variable, sometimes not administered and varied between 4 and 10 mg. By 2014, a more consistent dose of 10 mg was being administered and by 2019 a majority were receiving 10 mg dexamethasone. To evaluate the effect of a higher dose of dexamethasone, a cut point of 8 mg was chosen with one group receiving 0–6 mg and the other group receiving 8 or 10 mg.

In both the total group and the PTApre < 100 group, ears which received the greater dose of dexamethasone had significantly better HP. In the total group, mean HP for the < 8 group was 15 while the 8+ group mean was 36.4 with a *p*‐value of 0.0001. In the PTApre < 100 group, mean HP for the < 8 group was 18.1 while the 8+ group mean was 29.6 with a *p*‐value of 0.0044 (Table 5). Low frequency HP was also statistically significant with a higher dose of dexamethasone, with mean HP 14.8 in the < 8 group versus 33.0 in the 8+ group (*p* = 0.0006).

### 3.6 Time (Year of Procedure)

A significant correlation between HP and the year of implantation was identified although the correlation was small (Table 6). A higher correlation coefficient (closer to 1.0) indicates stronger correlation. The total group had a Spearman correlation coefficient, *r* = 0.22 (*p* < 0.0001), and the PTApre < 100 group also had *r* = 0.25 (*p* = 0.0002). The low frequency HP also had a small but significant correlation, *r* = 0.21 (*p* = 0.0001).

**TABLE 6:** Correlation between time (year of procedure) and hearing preservation.

Total group^a^	*r* = 0.22 (*p* < 0.0001)
PTApre < 100^a^	*r* = 0.25 (*p* = 0.0002)
Low frequency^a^	*r* = 0.21 (*p* = 0.0001)

^a^ All groups showed statistically significant correlation.

### 3.7 Multivariate Model

A linear regression model with a random patient effect was used to explore the effect of the following covariates: dexamethasone (< 8 and 8+), electrode type, sex, age at procedure, year of procedure and day of post measure. For the subset where PTApre < 100, none of the covariates were statistically significant. Dexamethasone dose and year of procedure were correlated (*r* = 0.70). Due to the correlation, separate models were run for dexamethasone dose and year of procedure. The effect of dexamethasone when adjusted for electrode type, sex, age at procedure and day of post measure was not significant (*p* = 0.0561). The effect of year when adjusted for electrode type, sex, age at procedure and day of post measure was not significant (*p* = 0.0869).

For low frequency HP, none of the covariates were statistically significant. Dexamethasone dose and year of procedure were also correlated (*r* = 0.75). The effect of dexamethasone when adjusted for electrode type, sex, age at procedure and day of post measure was significant (*p* = 0.0156). The effect of year when adjusted for electrode type, sex, age of procedure and day of post measure was also significant (*p* = 0.0443).

## 4 Discussion

This study examined the HP rate after cochlear implantation from 2010 through 2020. During this time, soft surgery techniques were instituted to minimize intracochlear damage and improve HP with an expectation that preservation would improve over time. We did find a small but significant improvement in HP over time on univariate analysis. This was true in all patients, those with preoperative PTA < 100 dB and low frequency HP. A higher dose of preoperative intravenous dexamethasone was significantly associated with better HP. Electrode type did not have an effect. Multivariate analysis across all frequencies did not show correlation with any of the investigated factors and overall HP although low frequency HP did show a correlation with dexamethasone dose and year of implantation.

Dexamethasone dose increased over time. However, separate models on multivariate analysis revealed that year of implantation was correlated with improved low frequency HP indicating that it was not only the increased steroid dose influencing results. This may indicate the benefit of soft surgical technique including changing from a cochleostomy to the round window approach and slower insertion speed.

While many studies on HP use the HEARRING method, many do not, which makes comparison across studies difficult. A comparison of studies from 2013 through 2021 showed a majority of studies reporting total plus partial HP rates near 50% with a few reporting rates above 80% [4, 5, 6, 7, 8, 9, 10, 11]. Newer studies continue to show complete plus partial HP between 36% and 90% [12, 13]. Preserved thresholds do appear to be stable over years [14, 15, 16, 17].

A temporal bone study showed that variations in surgical technique result in less trauma to hair cells within the cochlea. This “soft surgery” technique has become standard insertion technique [18]. A review of electrode design and surgical technique evolution for EAS stressed that mechanical damage, waves within the perilymph, acoustic trauma from drilling into the cochlea, loss of perilymph, fibrous tissue formation and possibly infection were factors in loss of residual hearing [19].

The entry point into the cochlea has changed from a cochleostomy to round window entry. With the newer thin electrodes, insertion through the round window minimizes the acoustic trauma of drilling into the cochlea as well as facilitates placement through a known landmark. An animal model reported that a round window approach resulted in less fibrosis than a cochleostomy [20]. There is conflicting data regarding whether this is a factor in humans. Several studies showed no difference in hearing change [9, 21, 22, 23]. Multiple other studies showed round window insertion to be superior to cochleostomy insertion for HP [8, 24, 25, 26, 27, 28]. Interestingly, Gotamco et al. found that insertion through an open round window versus a slit in the round window membrane resulted in higher complete HP [29]. Unfortunately, documentation regarding entry into the cochlea was not uniform at our institution limiting our ability to include this as a variable.

A slow rate of insertion has been postulated to improve HP as this may decrease trauma from the intra‐cochlear fluid wave during insertion. Studies have used different rates of insertion with slower insertion resulting in improved hearing outcomes [4, 30, 31]. Additionally, the choice of packing material for the cochlear opening has been shown to influence hearing results with the use of bone cement or fibrin glue leading to worse outcomes compared to tissue only or no seal [20].

Implantation results in fibrosis within the cochlea. A human temporal bone study in a patient that had initial HP but lost hearing over time showed intracochlear fibrosis and osteogenesis within the cochlea but no change in the number of spiral ganglion neurons between the implanted and non‐implanted ear [19]. This led to an animal study looking at the effect of steroids, which found that both systemic and topical steroids improved HP, although the systemic steroid dose would be very high when translated into a human dose [32]. Human studies have shown that steroids, both systemic and systemic plus topical, may improve HP but effects remain variable [24, 33, 34, 35, 36, 37, 38, 39, 40]. Recent systematic reviews have shown that perioperative steroid administration improves HP, but other types of administration or prolonged administration were not well studied [38, 39, 40, 41].

The type of electrode has also been shown to influence HP. An animal study showed that a stiff electrode produced more hearing loss compared to a soft electrode but that opening into the cochlea did not produce long term hearing loss [42]. Electrodes have become thinner and more flexible over time with the type of electrode grouping within two types: straight or perimodiolar. As with other variables, there is conflicting data on HP results. There are studies showing no difference while others show benefit with a straight array [4, 5, 6, 7, 8, 9]. Newer studies tend to favor straight and shorter electrodes [43, 44, 45, 46, 47, 48] with systematic reviews favoring straight and shorter arrays. While our institution offers implants from 3 manufacturers, we use primarily Cochlear and Advanced Bionics straight arrays while our PM arrays were the majority Cochlear which made analyses of specific arrays poorly powered due to the low number in each category.

New techniques in cochlear implantation such as intraoperative electrocochleography (ECOG) monitoring, robotic surgery, and other adjunct medications are being studied. Multiple studies have found that maintenance of ECOG values throughout insertion correlates with improved HP, and electrode pull back during insertion may improve ECOG values, although results are not uniform [49, 50, 51, 52, 53, 54]. Robotic surgery results have shown a tendency for improvement in low frequency hearing thresholds [55, 56, 57]. The addition of antioxidant *N*‐acetyl‐l‐cysteine has shown promise, although drug‐eluting electrode results are mixed [58, 59, 60].

EAS patients have better speech understanding than those using a cochlear implant alone [61, 62, 63]. None of our patients have elected to use EAS. Several studies have noted that usage of EAS is less than 50% and declines over time [11, 64]. However, Dalbert et al. showed that those with residual hearing have better CI speech understanding than those with no HP [14]. Therefore, HP remains a priority.

### 4.1 Limitations

One limitation of this study is that it is a retrospective review. Our institution did not systemically test postoperative residual hearing until 2018 so prior to 2018, some patients had a postoperative audiogram within weeks after implantation and some did not have an audiogram for more than a year after implantation. Most patients did not have more than one postoperative test so there was insufficient data to look at the stability of HP over time.

Another limitation is the lack of standardization of the surgical technique. While the three surgeons' operative techniques were similar, the transition for entry to the cochlea from cochleostomy to round window did not occur simultaneously, and the type of cochlear entry was not uniformly indicated. The transition from varied doses of intraoperative steroids to a set dose of dexamethasone also did not occur simultaneously. Additionally, our neurotology fellows perform electrode placement, which may impact the results. Therefore, date of insertion was used as a proxy to capture multiple elements of soft surgery.

Now our technique is more standardized with a slow insertion speed through the round window and a fixed perioperative steroid dose. All recipients who are programmed at our institution have at least one postoperative audiogram within 3 months of implantation. These changes have resulted in a more uniform approach to cochlear implantation surgery and audiometric testing, which should result in data with less variability moving forward.

Finally, our patients have preoperative thresholds that are closer to the profound range across all frequencies. It is unknown whether this is selection bias from the patients or whether this bias is from our own and referring audiologists. The high thresholds make measuring HP difficult as the preoperative thresholds are often at the limits of the audiometer leading to a floor effect, although this was attempted to be mitigated by use of the HEARRING model.

## 5 Conclusions

Cochlear implantation is the only procedure that can restore hearing but with the potential loss of residual hearing. Advances in surgical technique and electrode design have improved HP. Other studies have not looked at longitudinal results over time as a proxy for these multiple factors. This study showed low frequency HP did improve over time though the correlation was small. Standardized protocols will improve our ability to decipher specific reasons for improvement and allow us to hone in on those variables to better preserve residual hearing.

## Funding

The authors have nothing to report.

## Conflicts of Interest

The authors declare no Conflicts of Interest.

## Data Availability

The data that support the findings of this study are available on request from the corresponding author. The data are not publicly available due to privacy or ethical restrictions.
